# Long-Term Mortality following Acute Noninvasive Ventilation for Obesity-Related Respiratory Failure: A Retrospective Single-Centre Study

**DOI:** 10.1155/2023/5370197

**Published:** 2023-10-12

**Authors:** Aditya Krishnan, Paul Ellis, Pearlene Antoine-Pitterson, Amy Oakes, Bethany Jones, Alice Turner, Rahul Mukherjee

**Affiliations:** ^1^Institute of Applied Health Research, University of Birmingham, Birmingham, ENG, UK; ^2^Department of Respiratory Medicine, Heartlands Hospital (Part of University Hospitals Birmingham), Birmingham, ENG, UK; ^3^Institute of Clinical Sciences, University of Birmingham, Birmingham, ENG, UK

## Abstract

**Introduction:**

Determinants of long-term mortality following acute hypercapnic respiratory failure have been extensively studied in patients with chronic obstructive pulmonary disease. However, respiratory failure due to obesity has not been studied to the same extent. This retrospective survey aims to identify whether admission pH is associated with long-term mortality in patients requiring acute noninvasive ventilation (NIV) for obesity-related respiratory failure (ORRF).

**Methods:**

Records from April 2013 to March 2020 were accessed from a NIV quality database at an acute teaching hospital. Adults with hypercapnic ORRF requiring acute NIV were included. pH data were grouped by threshold (pH≤ and >7.25) and correlated with time from presentation to death; multivariable analysis was performed using Cox proportional hazards.

**Results:**

A total of 277 acute NIV episodes were included. Two-year mortality was similar for patients in both pH categories. Univariable analysis identified pH ≤ 7.25 to increase risk of two-year mortality by 43%. However, multivariable analysis identified that pH was not a significant determinant of long-term mortality, although male sex, older age, and higher admission pCO2 increased the risk of death at two years by 76%, 3% per year of age, and 16% per 1 kPa of pCO2 increase, respectively.

**Conclusion:**

Severity of hypercapnia on admission, male sex, and older age are associated with worse two-year mortality in patients requiring acute NIV for ORRF. There is scope for further analyses including investigating the role of domiciliary NIV in ORRF patients.

## 1. Introduction

Obesity is a global epidemic affecting over a quarter of the global population, with rising incidence and wide-reaching health ramifications [1]. Respiratory consequences of obesity include hypercapnic respiratory failure through impairment of pulmonary dynamics including decreased chest wall compliance, airway resistance and reduced lung volume, sleep-related breathing disorders such as obstructive sleep apnoea (OSA), and impaired respiratory drive [2, 3]. Furthermore, obesity is strongly associated with other drivers of morbidity such as hypertension and diabetes mellitus.

Obesity-related respiratory failure (ORRF) refers to the clinical phenomenon where obesity (body mass index (BMI) ≥ 30 kg/m^2^) is the primary cause of alveolar hypoventilation resulting in hypercapnia (pCO2 > 6 kPa), usually but not invariably associated with sleep-disordered breathing like OSA [3]. Up to a third of obese patients develop ORRF and may experience exacerbations resulting in acute hypercapnic respiratory failure (AHRF) requiring bilevel noninvasive ventilation (NIV) for hypoventilation due to the blunted hypercapnic ventilatory response. Development of AHRF is associated with worse outcomes including in-hospital mortality, and initiation of acute NIV is associated with improved outcomes [4]. Most of this evidence exists for chronic obstructive pulmonary disease (COPD), although ORRF is an area of increasing interest.

The evidence base for ward-based NIV is emerging, and recent reviews have supported its use based on cost effectiveness, and demonstrated noninferiority of ward-based NIV to critical care NIV for conditions such as ORRF [5]. Admission pH is a known predictor of survival to discharge for conditions other than COPD, with a pH under 7.15 significantly increasing the risk of in-hospital mortality [6, 7]. Ongoing pH of less than 7.25 while on NIV has been associated with worsened outcomes in COPD, but no similar evidence exists for ORRF [8].

In AHRF, multiorgan failure, age, and presence of consolidation on chest radiography are known to contribute to this risk of mortality [9, 10]. Prospective studies identified that when treated with the same protocols, ORRF patients requiring acute NIV for AHRF tend to have better outcomes than COPD patients for similar presentations [11, 12]. However, when adjusted for demographic confounders, there is no difference in rates of NIV failure, length of admission, or hospital readmission [5]. Consequently, British Thoracic Society (BTS)/Intensive Care Society (ICS) guidelines for initiation of NIV for ORRF do not differ from COPD, despite the recognition that ORRF patients with AHRF tend to respond better to treatment [4]. Research into factors affecting long-term mortality in COPD patients has identified age, cardio-cerebro-vascular comorbidities, admission pCO2, and previous hospitalisations for COPD exacerbations to be determinants of one-year mortality; the same evidence does not exist for ORRF patients [13, 14]. Indeed, the only research on long-term mortality for ORRF is on ambulatory patients using domiciliary NIV [15–17]. This retrospective survey aims to bridge this gap in knowledge by identifying the factors which affect one-year mortality in ORRF patients requiring acute NIV for AHRF.

## 2. Methods

### 2.1. Study Population

This is a retrospective observational cohort analysis based at a large acute hospital in West Midlands (England). Data were prospectively and routinely collected as part of the continuous audit of the physiotherapy-led ward-based NIV service and registered as an audit in the National Health Service institution's Clinical Audits and Registries Management Service (registration number: CARMS 16173). NIV was managed using BTS/ICS guidelines with regular input from consultants of Respiratory Medicine [4]. Patients requiring acute NIV for ORRF were identified from the audit database.

The analysis included all adult patients requiring acute NIV for ORRF as the physician-determined primary indication. Where the primary indication was multifactorial, ORRF was identified as the predominant cause when (a) COPD, even if present, was not severe enough to cause respiratory failure (forced expiratory volume in 1 second (FEV1) > 50% predicted), (b) there was no current or prior diagnosis of neuromuscular weakness, and (c) BMI > 40 kg/m^2^. Patients admitted to the hospital between 01 April 2013 and 09 March 2020 were included. The start date was selected to correspond to the routine recording of full arterial blood gas (ABG) results on the database. The end date was selected to exclude changes to the service caused by the COVID-19 pandemic, as the first COVID-19 patient requiring NIV was admitted on 10 March 2020. Finally, only the most recent episode of acute NIV recorded on the database per patient was used to avoid immortal time bias. Records were excluded if they were missing data on admission date, date of birth, or ABG results prior to initiation of NIV (details in Table 1).

### 2.2. Analysis

Statistical analysis was performed using R version 4.1.2 and the following packages: “tableone” [18], “survival” [19], and “survminer” [20]. Statistical significance was set at *p* < 0.05. The primary endpoint for long-term mortality was the number of deaths at one year. One year was selected as the endpoint to correspond with the outcomes frequently reported for long-term mortality in COPD [21, 22]. Escalation to ITU was intended as a secondary outcome but not performed due to absent data. Treatment success was defined by cessation of NIV due to resolution of AHRF without the need for invasive mechanical ventilation or survival to discharge with the setup of domiciliary NIV. Patients with early NIV withdrawal without clinical improvement, insufficient resolution of AHRF, those requiring invasive mechanical ventilation, or inpatient death were considered treatment failures.

Univariable analysis was first performed to identify determinants of long-term mortality, by grouping data by thresholds for admission pH. Initial analysis set a pH threshold of 7.35 to correspond with the BTS/ICS definition of AHRF as an indication for initiation of NIV [4]. However, the BTS 2013 NIV audit demonstrated that over 40% of AHRF patients with a pH under 7.25 requiring NIV are managed on a ward setting [23]. This is despite half these patients having a care plan which includes invasive ventilation. As a result, a pH threshold of 7.25 was picked to reflect the experiential realities of ward-based NIV practice in the UK, where a large proportion of patients undergoing ward-based NIV are more acidotic than BTS/ICS guidelines.

Grouped data were correlated with time from presentation to death using the log-rank test, and Kaplan–Meier curves were generated. Subsequently, Cox proportional hazards model was used to perform a multivariable analysis of mortality using the following variables: age, sex, BMI, admission pH, and admission pCO2. Schoenfeld residuals were calculated to ensure stability of the hazard. Other variables such as presence of consolidation on chest radiography and clinical frailty score could not be included in the analysis, as the studied dataset only collected variables suggested by the BTS audit standards.

## 3. Results

A total of 277 acute NIV episodes were included in this analysis, consisting of the most recent NIV episode for each unique patient; 45% of these episodes presented with an admission pH ≤ 7.25.  Table 2 demonstrates the baseline admission and outcome characteristics split by admission pH thresholds: ≤7.25 and >7.25. Median pH for the entire studied population was 7.26. Median pCO2 expectedly and significantly varied across groups, as did mean admission age. Over half of all patients in this cohort experienced multiple distinct episodes of NIV. Patients with an admission pH ≤ 7.25 had a median of two episodes in the study period, significantly different to only one admission for pH > 7.25 (*p*=0.006).

On average, patients required almost 16 hours of acute NIV spread over at least two days, with an average hospital stay of almost two weeks. The majority of patients in this cohort successfully completed their episode of NIV, with a subgroup going on to have domiciliary NIV set up on discharge. Thirty-three patients required escalation to intensive care, and 22 patients died in hospital. Rates of these inpatient outcomes did not vary significantly across either pH threshold. Number of deaths significantly varied across the pH threshold at both one and two years. Finally, there were no statistically significant differences between the average number of days until death, BMI, or patient sex in a univariable analysis between the two pH thresholds.

**Figure 1:**
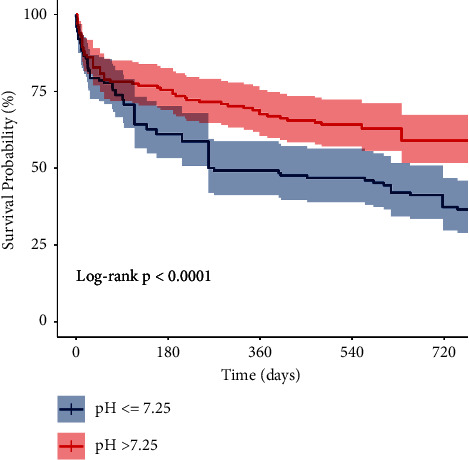
Kaplan–Meier survival curve at two years with admission pH threshold of 7.25 (95% confidence intervals highlighted in colour).

Figure 1 shows a Kaplan–Meier curve comparing two-year mortality for either pH threshold group and demonstrates that both groups follow a similar trajectory for the first few months, but with statistically significant difference in mortality at one and two years. Univariable analysis identifies an admission pH of ≤7.25 to increase risk of two-year mortality by 43% compared to pH >7.25 (RR: 1.43, 95% CI: 1.18–1.74, log-rank *p* < 0.0001). Two-year mortality for the entire studied cohort was 52% (*N* = 143).

**Figure 2:**
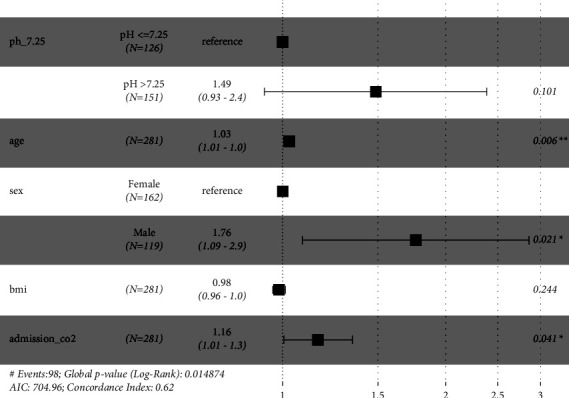
Cox proportional hazards model.

Cox proportional hazards model was used to perform multivariable analysis between these pH subgroups (Figure 2), with Schoenfeld residuals calculated (Table 3). The analysis demonstrated that admission pH and BMI were not determinants of one-year mortality. Male sex was associated with a 76% increased risk of mortality at one year compared to female sex (RR: 1.76, 95% CI: 1.09–2.90, *p* = 0.021). Although a higher pH on admission was associated with a better survival in the studied population, this was not statistically significant (RR: 1.49, 95% CI: 0.93–2.4, *p* = 0.101) unlike the univariable analysis using log-rank test. For this cohort, every additional year of age was associated with a 3% increased risk of one-year mortality (RR: 1.03, 95% CI: 1.01–1.0, *p* = 0.06), and every additional kPa of pCO2 was associated with a 16% increased risk (RR: 1.16, 95% CI: 1.01–1.3, *p* = 0.041).

## 4. Discussion

This study demonstrates that in ORRF patients requiring acute NIV for AHRF, admission pH is not a determinant of long-term mortality based on multivariable analysis. National audit data estimate the median admission pH for patients who received acute NIV to be 7.24, which was comparable to 7.26 in this studied ORRF population [23]. Median age was statistically different across groups; the chronic and progressive nature of many respiratory conditions results in an expected deterioration of the disease with age, resulting in worse acidosis during AHRF. Furthermore, older patients are more likely to be comorbid, resulting in a greater metabolic component contributing to acidosis. pCO2 was expectedly different across groups, as it is the major determinant of pH in this population with a respiratory presentation. Around half the patients in either group had experienced previous hospital admissions requiring NIV, although patients with a lower pH had a greater number of median admissions.

Multivariable analysis demonstrated that males, older patients, and worse hypercapnia all increased the risk of one-year mortality. Cross-sectional studies have identified male sex to be associated with greater cardiometabolic comorbidities as well as obesity-related conditions such as musculoskeletal disorders [24, 25]. Similarly, increased age is associated with increased comorbidity burden and generally poorer physiological reserve [26]. Furthermore, worse hypercapnia on admission represents a greater severity of the underlying respiratory condition. BMI had been hypothesised to be a determinant of mortality due to associated comorbidities and the physiological effect of obesity on metabolic and respiratory systems, although the results for this were not statistically significant. A previous retrospective analysis on a similar dataset identified admission pH < 7.15 to be significantly associated with increased in-hospital mortality in ORRF patients needing acute NIV [9]. However, other prospective and retrospective studies have not identified pH as a determinant, instead pointing to comorbidity burden as a greater determinant [10, 27]. While the univariable analysis refutes this finding by identifying that admission pH of < 7.25 increased risk of two-year mortality by 43%, multivariable analysis does not identify increased risk due to low admission pH.

The 2019 BTS national NIV audit demonstrated a 76% rate of NIV success, similar to 74% in the studied population [28]. The national audit patient population was 56% female, similar to 57% in this studied cohort. The national median age was 72 compared to 67 years in this study—this can be attributed to the relatively young population serviced by the studied hospital, with a high degree of social deprivation resulting in poorer health at a younger age [29].

A greater proportion of patients presenting with pH ≤ 7.25 had previous episodes of acute NIV compared to pH > 7.25; additionally, they were also more likely to have a greater number of previous acute NIV episodes. This likely reflects the worse severity of disease in highly acidotic patients, with serially progressive disease on each subsequent admission. Analysing the progression of acidosis and hypercapnia for each unique patient on consecutive admissions for acute NIV fell beyond the scope of this study but can be investigated in a future analysis. Univariable analysis identified that the patients admitted with a lower pH were at 43% higher risk of death at two years, with a statistically significant greater proportion of patients dying; however, this effect was not observed in the Cox proportional hazards model. This effect was hypothesised as severe acidosis does correlate with in-hospital mortality; it is contributed to by worse baseline respiratory disease, severe metabolic comorbidities, and acute hypercapnia. There is no existing evidence investigating whether admission pH is a determinant of long-term mortality for ORRF. Data from the multivariable analysis identify that there is no long-term impact of admission pH in this cohort, but pH at presentation likely represents a greater comorbidity burden and readmission risk. However, prospective clinical trials are necessary prior to changing clinical practice.

An advantage of this study is the ability to design a protocol considerate of potential biases. For instance, the missingness of data could be analysed prior to writing a statistical analysis strategy, to ensure that appropriate variables were being considered. This study analyses a large patient population with detailed data compared to similar previously published research and presents conclusions previously not available for ORRF patients requiring acute NIV for AHRF. As these data are routinely collected as part of the acute NIV service, they are not subject to participation or attrition bias and the results may be more externally valid. The design of this study allowed us to set clear dates for inclusion and exclusion to account for known historic temporal confounders, such as COVID-19 from March 2020.

The limitations of this study are its retrospective observational cohort design and ORRF being physician-determined as the primary indication for NIV, although we have defined in the *Methods* section how we arrived at ORRF as the primary cause of AHRF. Furthermore, the most acutely unwell patients presenting with low GCS, apnoea, or cardiac arrest would have been contraindicated for acute NIV and therefore would not be included in the database on which this analysis was performed. Observational studies carry limitations, such as conclusions reliant on the strength of the prospective data collection, variables which are routinely collected, and confounding factors. Future data collection will aim to include a record of comorbidity burden and clinical frailty, which was not categorically defined in the current dataset.

This study builds on existing work to identify determinants of long-term mortality in ORRF patients requiring acute NIV for AHRF. Obesity itself has emerged as a strong risk factor for the requirement of intubation or death in viral respiratory infections like H1N1 influenza and COVID-19 [30, 31]. It is also important to include a comorbidity index in future scientific surveys (such as the Charlson Comorbidity Index) to ascertain the role of comorbidities in determining prognosis. Evidence from COPD patients demonstrates that patients presenting with acute-on-chronic hypercapnia who are initiated on home mechanical ventilation have improved admission-free survival [32]. This is an area for further study in ORRF, including in those patients with ORRF without severe obstructive sleep apnoea [33]. There is a role for further analysis focussing specifically on the effectiveness of domiciliary NIV following admission with AHRF due to obesity.

## Figures and Tables

**Table 1:** Missingness of data for each variable and group.

	pH ≤ 7.25	pH > 7.25
*n*	126	151
Age	126 (100%)	151 (100%)
Gender	126 (100%)	151 (100%)
BMI	66 (52%)	81 (54%)
Admission pH	126 (100%)	151 (100%)
Admission PaCO2	126 (100%)	151 (100%)
Admission bicarbonate	126 (100%)	151 (100%)
No. With previous admissions	126 (100%)	151 (100%)
Length of admission	126 (100%)	151 (100%)
Length of acute NIV use	126 (100%)	151 (100%)
Total no. of acute NIV episodes	126 (100%)	151 (100%)
Outcome at end of NIV episode	121 (96%)	150 (99%)
Escalation to ITU	121 (96%)	146 (97%)
Mortality status at two years	126 (100%)	151 (100%)

Values in parentheses represent the proportion of data present for that variable, as a proportion of the total number of patients included in that group.

**Table 2:** Baseline and outcome characteristics of included patients by pH threshold.

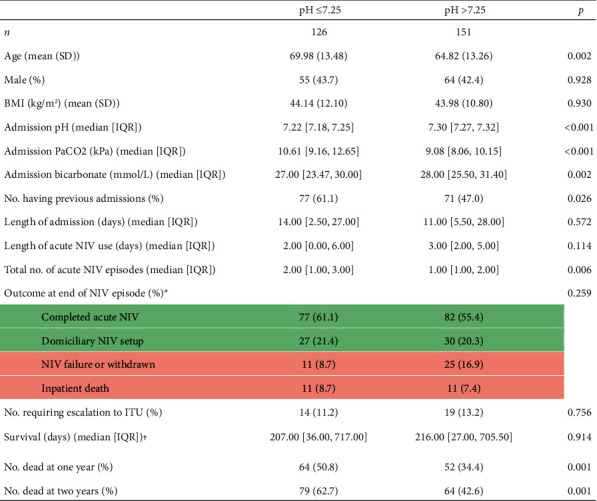

^*∗*^Outcome is defined as a categorical variable and patients can only be included in one category, i.e., domiciliary NIV setup implies completion of acute inpatient NIV episode, and inpatient death implies NIV failure. ^†^Of patients who died within two years. SD: standard deviation; BMI: body mass index; PaCO2: arterial partial pressure of carbon dioxide; kPa: kilopascals; mmol/L: millimoles per litre; NIV: noninvasive ventilation; IQR: interquartile range; ITU: intensive treatment unit.

**Table 3:** Schoenfeld residuals for Cox proportional hazards model.

	Chi-squared	*p*
pH	0.2128	0.645
Age	1.1421	0.285
Sex	1.6094	0.205
BMI	5.0590	0.024
Admission pCO2	0.0592	0.808
Global	**8.8225**	**0.116**

Bold values demonstrate the values for the entire dataset.

## Data Availability

Access to data is restricted due to institutional data protection regulations.
